# Characterization of Olanzapine-Solid Dispersions

**Published:** 2011

**Authors:** Venkateskumar Krishnamoorthy, Arunkumar Nagalingam, Verma Priya Ranjan Prasad, Siva Parameshwaran, Neema George, Punitha Kaliyan

**Affiliations:** a ***Department of Pharmaceutics, KMCH College of Pharmacy, Coimbatore, India.***; b ***Department of Pharmaceutical Sciences, BITS, Ranchi, India.***

**Keywords:** Pregelatinised starch Sodium starch glycollate, X ray diffraction, Near infra red spectroscopy, Differential scanning calorimetry, Fourier transform infrared spectroscopy.

## Abstract

Aim: To enhance the aqueous solubility of olanzapine by using the Solid dispersion technique. Solid dispersions of olanzapine were prepared by the dispersion method using using PGS and SSG as carriers. Drug-carrier ratios such as 1 : 1, 1 : 2, 1 : 4, 1 : 6, 1: 8 and 1 : 10 were tried for optimization. Characterization was done by phase solubility, in-vitro release, saturation solubility, permeation, wettability, XRD and FTIR analysis. Solid dispersions showed higher solubility and an improved drug release profile than the pure drug. Solid dispersion and physical mixture with a drug-polymer ratio of 1 : 10 showed the best release profile in comparison with the other samples. Phase solubility results verified the solubilization effect of the carrier. XRD and NIR analysis confirmed the reduction of crystallinity in the samples. The release study findings were well supported by the results of wettability, saturation solubility and permeability studies. IR analysis substantiated the inertness of the carrier. It was concluded that pregelatinised starch (PGS) and sodium starch glycollate (SSG) could be utilized as effective carriers to improve the aqueous solubility of poorly soluble drugs.

## Introduction

Solubility is an important physicochemical factor affecting the absorption of drugs and their therapeutic effectiveness. Poor aqueous solubility leads to formulation development failures. The poor solubility of drug substances in water and their low dissolution rate in aqueous G.I.T fluid often leads to insufficient bioavailability, and an increase in dosage and variability in blood concentrations (1). About 35- 40 % of drugs suffer from poor aqueous solubility. The formulation of poorly soluble drugs for oral delivery represents one of the major challenges to formulation scientists in the drug industry (2-6) Solubility enhancement can be achieved by increasing the surface area of the drug which is accessible to the dissolution medium.

The solid dispersion is the most commonly used technique for improving the dissolution and bioavailability of poorly soluble active pharmaceutical ingredients as it is simple, economic and advantageous. The term solid dispersion was defined by Chiou and Rigelmann as “The dispersion of one or more active ingredients in an inert carrier or matrix at solid state melting, solvent or melting solvent method.” (7). It can be used to increase the dissolution rate of the drug with low aqueous solubility, thereby improving its oral bioavailability. Higher drug dissolution rates from a solid dispersion can be facilitated by optimizing the wetting characteristics of the compound’s surface and also by increasing the interfacial area available for drug dissolution (8). A wide variety of water soluble carriers have been used for enhancing the aqueous solubility of drugs. Several insoluble drugs have been shown to improve their solubility, dissolution rate and oral absorption when incorporated into a solid dispersion. (9, 10).

The objective of this work is to improve the aqueous solubility of olanzapine an atypical antipsychotic drug, by using the solid dispersion technique utilizing pregelatinised starch (PGS) and sodium starch glycollate as carriers. Due to its poor solubility its bioavailability is greatly affected in vivo. This factor perfectly meets the requirments for the objective of this work. 

## Experimental

*Material and methods*

Olanzapine was obtained as gift sample from M/s. Orchid Chemicals, Chennai., Pregelatinsed starch (PGS) and sodium starch glycollate was obtained from M/s.Colorcon Limited, India. All other solvents and reagents used were of analytical grade.

*Phase solubility study*

Drug and carrier as per specific drug-carrier ratio were weighed accurately and added to 25 mL of water in screw capped bottles, then shaken in a remi orbital incubator shaker at 37°C and 24°C for 24 h (10).The container containing the pure drug and water alone was used as a control. After 24 h the solutions were filtered, diluted and the absorbance levels were measured at 250 nm (11).

*Preparation of solid dispersions*

Solid dispersions with different drug-carrier contents was prepared by using the dispersion method. Olanzapine was dissolved in acetone and a specific amount of powdered carrier was placed in a mortar. The drug solution was slowly added to the powdered carrier with constant trituration till a porous mass was formed. The mass was then dried in vaccum oven maintained at -1 kg/cm^2^ at room temperature, pulverized and passed through sieve No.-80 to get uniform sized particles (12).

*Assay of solid dispersions*

Solid dispersions equivalent to 50 mg of drug were weighed and dissolved in 50 mL of 0.1 N hydrochloric acid, filtered, diluted and the absorbance levels were measured at 250 nm. 

*In-vitro release studies*

*In-vitro *release studies were performed in USP XXIX Dissolution rate (paddle type) apparatus (Electrolabs, Mumbai) using 900 mL of 0.1 N HCL as the dissolution medium at 37°C. An amount of solid dispersion equivalent to 20 mg of olanzapine was added to the dissolution flask and samples were withdrawn at predetermined time intervals maintaining sink conditions at each time interval. Studies were conducted for a period of 1 h with the above fixed parameters in triplicate. The withdrawn samples were diluted and the absorbance levels were determined at 250 nm. The average amount of olanzapine released was then calculated from the recorded values. 

*Saturation solubility analysis*

Weighed amount of olanzapine (pure drug) and solid dispersions equivalent to 20 mg of drug were added to 10 mL of distilled water in 25 mL stoppered conical flasks. The flasks were then agitated on a rotary shaker for 24 h at 27°C and equilibrated for 2 days. An aliquot was filtered, then diluted and analyzed at 250 nm (13).

*Wettability study*

Pure drug and selected formulations of about 50 mg were weighed and placed in a Buchner glass funnel. Methylene blue powder (50 mg) was layered uniformly on the surface of the powder in the funnel and plunged into a beaker containing water at the same level as the powder. The time required for wetting the methylene blue powder was taken as the wetting time. 

A tissue paper was placed in a petri dish with a diameter of 10 cm. Methylene blue, a water soluble dye, was added to the petri dish. A tablet compressed from the selected batch was carefully placed on the surface of the tissue paper and the dye solution was used to distinguish when complete wetting of the tablet surface had occurred. The time required for water to reach the upper surface of the tablets and completely wet their surface was taken as the wetting time. The weight of the tablet was noted before (w_b_) and after the study period (w_a_). From the data, water absorption ratio R, was calculated as using the following equation. (14, 15).

R = 100* (w_a_- w_b_) / w_b_

**Table 1:** Phase solubility data

**Carrier**	**Temp** **°C**	**Slope**	**Intercept**	**Ka**	**ΔG** **kJ/mol**	**ΔH** **kJ/mol**	**ΔS** **kJ/mol**
**PGS**	25	33.800	-0.787	1.310	-9.184	-9.184	-9.153
	37	37.359	-0.474	8.746	-22.54	-22.54	-22.47
**SSG**	25	534.63	-19.430	0.0511	-2.608	-2.6086	-2.599
	37	662.53	-24.057	0.0416	-2.686	-2.6869	-2.678

*Permeation study*

The permeation study of the pure drug and solid dispersions were carried out using egg and cellulose nitrate membranes. The diffusion of the drug through the membranes was analyzed in a diffusion cell and the amount of drug permeated in a given period of time was determined at 250 nm (16, 17).

*X-ray diffraction study*

All the selected formulations and pure drugs XRD patterns were recorded on a PW1729, Philips diffractometer (Eindhoven, The Netherlands) using Ni-filtered, CuKα radiation, with a voltage of 40 kV and 25-mA current. The scanning rate employed was 10 min^-1^ over a 10 to 30^0 ^diffraction angle (2θ) range. The analysis was performed at the central electrochemical research institute, Karaikudi. 

*Near infra red analysis*

The near infra red spectra of the pure drug, carrier and selected batch was analyzed in a double beam FTIR spectrophotometer (Shimadzu, Japan) using the KBr pellet technique. Various parameters such as the absorbance, FWHM, peak intensity and peak base were compared among the spectra.

*Drug-polymer interaction analysis*

The IR spectrum of the pure drug, solid dispersions and physical mixtures were analyzed in a double beam FTIR spectrophotometer (Shimadzu, Japan) using KBr pellet technique.

## Results

*Phase solubility study*

Table1. Gives the phase solubility and the thermodynamic parameters of the samples at two different temperatures. 

*In-vitro release studies*

**Figure 1:**
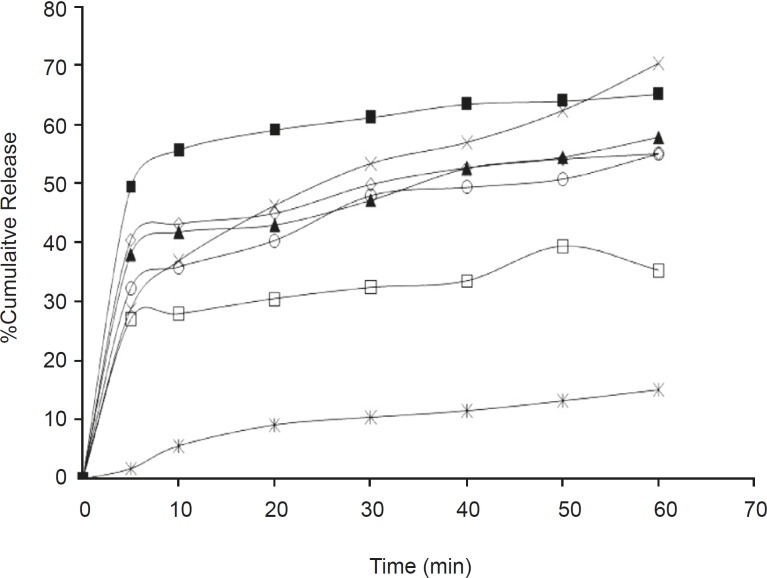
*In-vitro *release profile of olanzapine –PGS solid dispersions with pure drug. Ж –Olanzapine, □ –OPGS1, ◊ - OPGS2, ○-OPGS4, ▲-OPGS6, ■-OPGS8, X – OPGS10

The dissolution parameters of the samples and profiles compared with the pure drug are shown in Table 2, 3 and Figure 1, 2 respectively. 

**Figure 2:**
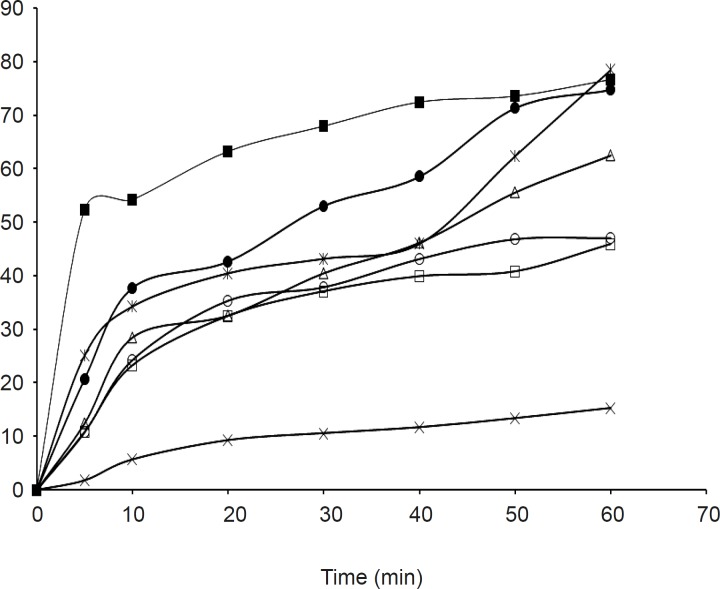
*In-vitro *release profiles of olanzapine-SSG solid dispersions with pure drug x- Olanzapine, □-OSSG1, ○- OSSG2, Δ-OSSG4, ●-OSSG6, ■-OSSG8, ж - OSSG10

**Table 2:** *In-vitro *dissolution parameters for olanzapine-PGS solid dispersions

**BATCH**	**CR05**	**CR30**	**CR60**	**DE**	**MDT**	**T50**
**1 : 0**	46.70 (0.60)	60.77 (0.39)	71.89 (0.58)	59.61	11.47	12.5
**1 : 1**	49.43 (1.20)	61.17 (2.18)	70.84 (2.89)	59.94	10.52	6.0
**1 : 2**	50.48 (1.36)	62.86 (1.63)	77.11 (2.36)	61.46	13.31	5.0
**1 : 4**	59.74 (1.41)	69.27 (3.19)	81.69 (3.53)	68.35	11.06	4.5
**1 : 6**	60.26 (2.78)	67.97 (2.78)	85.60 (3.26)	67.85	13.57	4.25
**1 : 8**	75.39 (1.41)	84.70 (3.19)	91.63 (3.53)	82.09	7.70	3.5
**1 : 10**	75.91 (2.36)	89.53 (1.18)	98.04 (1.63)	86.24	8.61	3.0

%CR: Cumulative release, % DE: Dissolution efficiency, MDT: Mean dissolution time

*Saturation solubility studies*

Table 3. Shows the saturation solubility data for olanzapine and selected batches of solid dispersions. 

**Table 3:** *In-vitro *dissolution parameters for olanzapine-SSG solid dispersions

**BATCH**	**CR05**	**CR30**	**CR60**	**DE**	**MDT**	**T50**
**1 : 1**	48.13 (2.35)	58.43 (3.42)	68.88 (0.45)	58.07	10.70	6.5
**1 : 2**	62.87 (1.58)	73.46 (1.85)	83.39 (0.60)	72.06	9.5	4.5
**1 : 4**	66.65 (1.20)	73.98 (1.26)	89.40 (2.96)	73.94	11.60	4.5
**1 : 6**	74.61 (1.58)	86.53 (1.38)	93.85 (1.26)	83.30	8.17	4.0
**1 : 8**	76.57 (1.63)	82.22 (1.20)	93.58 (0.60)	80.98	9.44	3.8
**1 : 10**	89.09 (0.55)	94.51 (2.22)	102.09 (1.10)	92.35	7.21	2.5

%CR: Cumulative release, % DE: Dissolution efficiency, MDT: Mean dissolution time

*Wettability studies*

The wettability time and water absorption ratio data is shown in Table 4. 

**Table 4:** Evaluation parameters for formulations

Formulation	Saturation Solubility (mg/mL)	Permeability (mg/mL/h)		Wettability	
		**Egg Membrane**	**Cellulose Nitrate Membrane**	**Buchner Funnel Method (Min)**	**WaterAbsorption Ratio**
**Pure Drug**	0.0278	.0030	.0137	80	3.904
**OPGS10**	0.0329	.0061	.0233	35	7.48
**OSSG10**	0.0323	.0055	.0227	49	6.65

*Permeation studies*

The permeability data for passing through egg and cellulose nitrate membrane is shown in Table 4. 

*XRD analysis*

**Figure 3:**
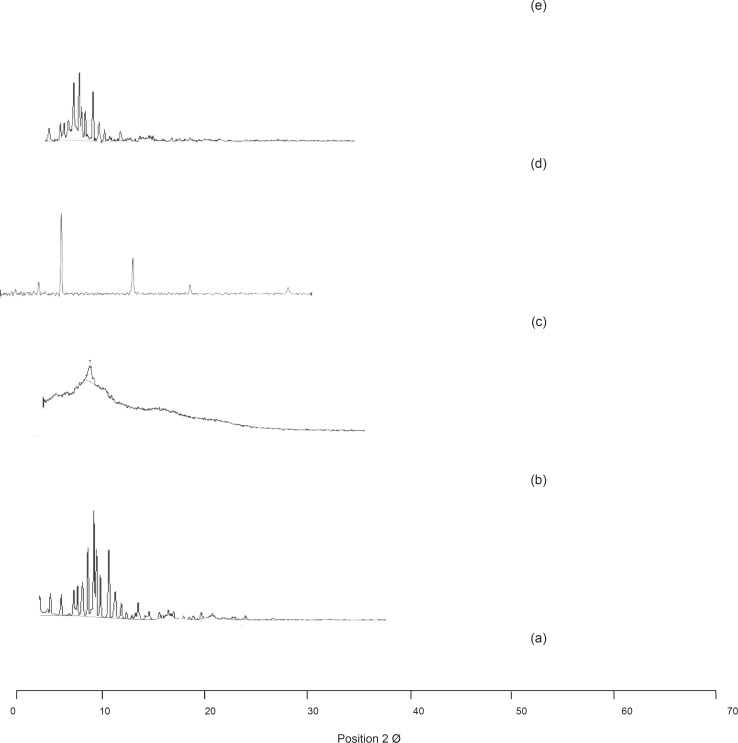
XRD spectra: (a) Olanzapine, (b) PGS, (c) SSG, (d) OPGS10, (e) OSSG10

Figure 3. Shows the comparison of the XRD spectra of olanzapine and its selected formulations. 

*NIR analysis*

**Figure 4:**
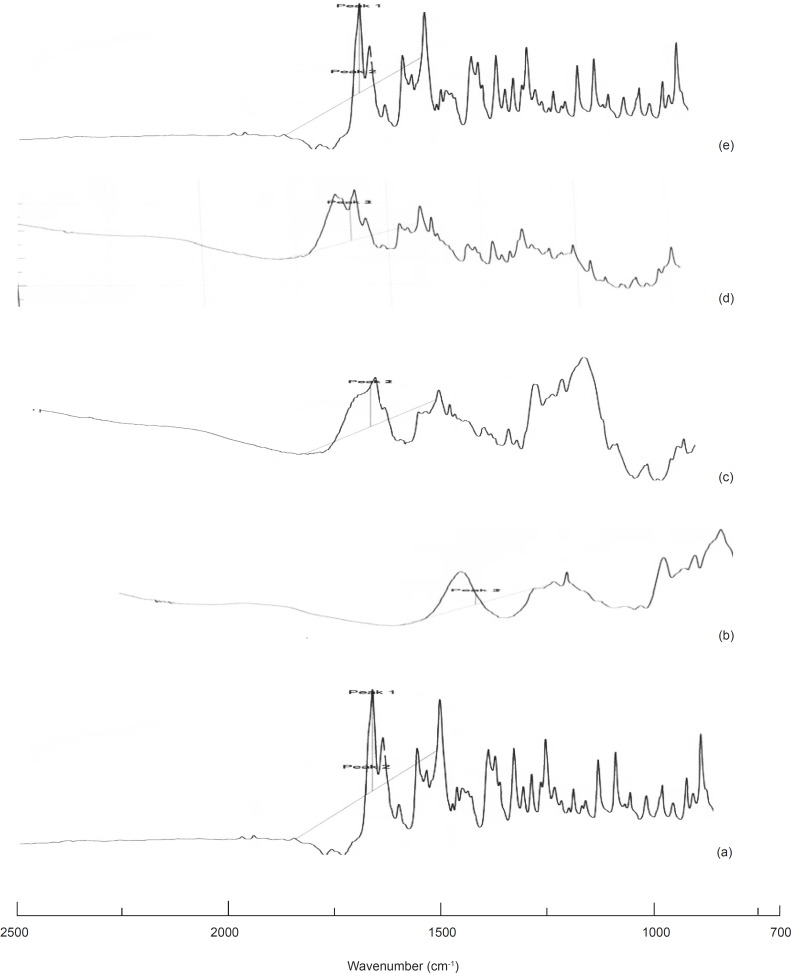
NIR peak height spectra: (a) Olanzapine, (b) PGS, (c) SSG, (d) OPGS10, (e) OSSG10

**Figure 5:**
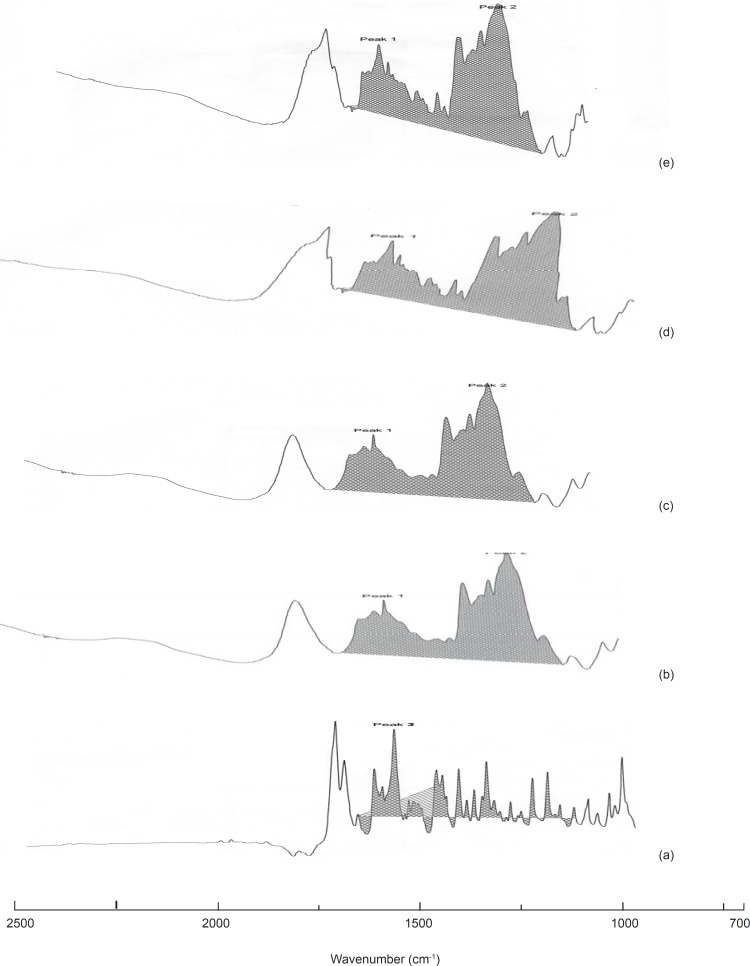
NIR peak area spectra: (a) Olanzapine, (b) PGS, (c) SSG, (d) OPGS10, (e) OSSG10

Particle size analysis-particle size analysis and FWHM values are shown in Table 5. Figure 4 and 5 shows the NIR peak height and NIR peak area spectra respectively. 

**Table 5:** NIR data

**Sample **	**Absorbance at specific wavelength nm **						**FWHM **
	**745.32 **	**1009.55**	**1223.61**		**1412.6**	**1585.20**	
**Pure drug **	1.2853	1.00461	1.1321	1.6621		1.7715	18.492
**OPGS10 **	0.6350	0.6448	0.6574	0.7883		0.6087	13.64
**OSSG10 **	0.8843	1.1940	0.9226	1.0703		1.1190	15.62

*DSC analysis*

**Figure 6:**
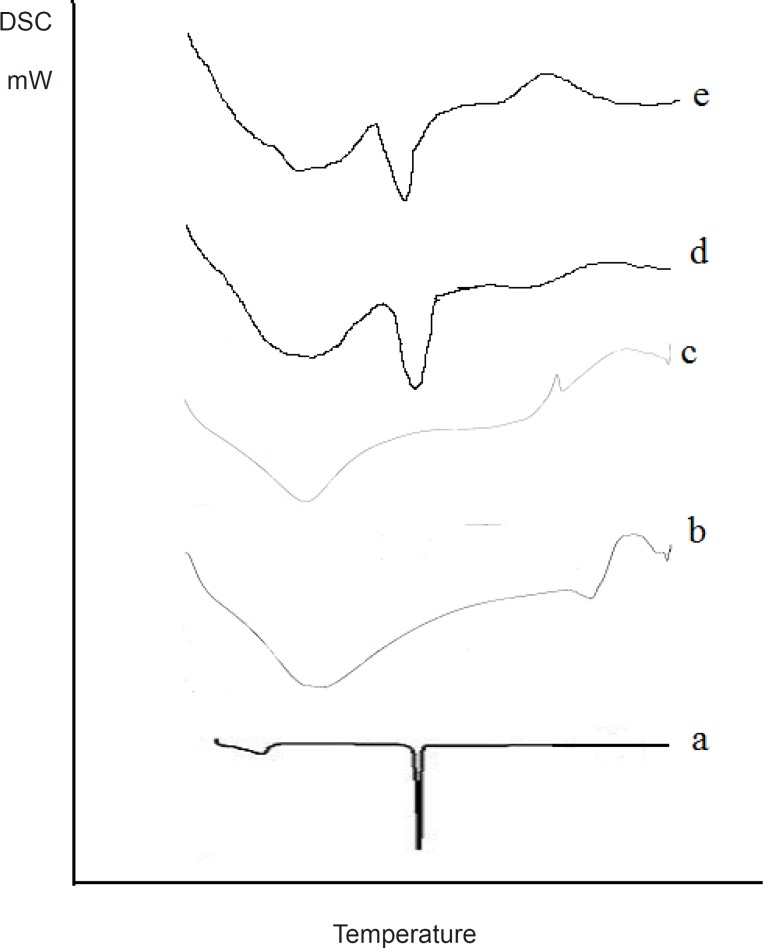
DSC spectra: (a) Olanzapine, (b) PGS, (c) SSG, (d) OPGS10, (e) OSSG10

The DSC spectra of the samples in comparison with the pure drug are illustrated in Figure 6. 

## Discussion

The negative ΔG, ΔH and ΔS values of the formulations indicate the spontaneity of the process at low temperature. The solubility of olanzapine was found to increase constantly on increasing the concentration of the carriers when physically mixed with the drug. These findings clearly verify the solubilization effect of carriers on the drug. 

*Assay data*

The drug content in the samples was found to be in the range of ± 5 % of the theoretical amount and a low C.V indicates the suitability and reproducibility of the method used for formulation. 

*In-vitro release studies*

The release rate from the solid dispersions was found to be much higher than that of the pure drug. In addition it was also noticed that batches OPGS10 and OSSG10 showed a higher cumulative release, better dissolution efficiency and less mean dissolution time; laying claim to being superior among its respective formulations. The release rate from the dispersions was found to be significantly different between the samples at a 5% level (p > 0.05) in comparison to the pure drug due to the effect of the carrier and its concentration. It was also observed that the release rate tends to increase as the concentration of the carrier increases, in both of the examined formulations. The findings of the release study are well supported by related works utilizing PGS and SSG as carriers, which showed an increase in solubility and dissolution rate in solid dispersions (21, 22). 

*Effect of carrier on release*

From the release study data it was also observed that PGS was found to be a better carrier than SSG for the improvement of the aqueous solubility of the model drug. 

*Saturation solubility studies*

The saturation solubility of the samples was found to be higher than that of the pure drug (p > 0.05) proving the efficiency of the carriers in enhancing the aqueous solubility of olanzapine. 

*Wettability studies*

The wetting time of the pure drug was found to be 80 min and the water absorption ratio 3.904, which clearly indicates its poor wettability. The wetting time of the selected samples was found to be much less and the water absorption ratio was higher than for the pure drug. (p > 0.05). This behavior may be attributed to an increased wettability due to the presence of hydrophilic carriers in the samples. 

*Permeation studies*

It was noted that, the amount of drug that permeated from the selected batches in both membranes was found to be higher than the pure drug (p > 0.05) and the permeation rate through the cellulose nitrate membrane was greater than that of the egg membrane. These findings can be considered as being evidence for an increased release rate of olanzapine from solid dispersions. 

*XRD analysis*

The XRD pattern of olanzapine showed numerous sharp, narrow and intense peaks, claiming its high crystallinity. The patterns of the carrier showed no peaks at all or only small ones indicating its amorphous nature. It was observed that, the number and intensity of the peaks were found to be less in the samples and the relative intensity percentage values were also found to show a decline phase. The bases of the peaks in the samples were also found to be broader in nature confirming a reduction in crystallinity. The decreased FWHM value of the intense peaks in the samples than those of the pure drug also confirms the reduction in the crystallinity of the tested samples. These conclusions from XRD analysis can be taken as confirmation for the reduction of crystallinity and phase transition (from crystalline to an amorphous form) in the samples. The observations were reported by the Center for Computational Project guidelines (19, 20).

*NIR analysis*

The absorbance values of the samples at specific wave lengths increase linearly with the particle size of the drug. It was observed that the absorbance value of the selected samples at specific wavelengths were found to be lower than that of the pure drug indicating that size reduction had occurred in the samples. 

Numerous sharp narrow peaks comprising of less area and high FWHM values were observed for the pure drug, indicating its high crystallinity. In comparison, it was found that the sample peaks were less in number, with a higher area, low FWHM values and broader in nature which clearly confirms that a reduction in crystallinity and phase transition had occurred in the samples (19, 20).

*DSC analysis*

A sharp narrow peak was obtained from the DSC spectra of Olanzapine indicating the high crystallinity of the drug. Broad Endothermic peaks were obtained for the carriers proving the amorphous nature of the carriers. When comparing the DSC spectra of the tested samples it was revealed that there was a shift in the glass transition temperature and the peak was found to be broader in nature. This confirmed phase transition and crystallinity reduction had occurred in the samples, this would have assisted in increasing the release rate from the tested samples.

*Mechanism of release*

Based on all of the above findings, the possible mechanisms for an increased release rate from solid dispersions have been postulated and are summarized as follows: (i) reduction of crystallinity (ii) increased water absorption by the hydrophilic carrier which leads to increased wettability(iii) solubilization effect by the carrier (iv) phase transition from crystalline to amorphous nature. The suggested mechanisms for the increased release rates were found to correlate well with related research works utilizing PGS and SSG as carriers (21, 22).

*IR studies*

Upon comparing the IR spectra of the samples with that of the pure drug, it was noticed that the characteristic peaks of the pure drug were also present in the sample spectra revealing the inert nature of the carrier used for formulation.

## Conclusion

The dispersion method used to formulate solid dispersions of olanzapine was found to be suitable and reproducible in nature. It was observed that Batch SD10 for both formulations with carriers PGS and SSG showed a marked increase in aqueous solubility and dissolution parameters than that of the pure drug. These factors meant that it was the best releasing batch from among the tested samples. The possible mechanisms for an increased release rate from the samples were also postulated and were well supported by study findings, such as XRD analysis, NIR analysis, *in-vitro *release, phase solubility, saturation solubility, wettability and permeation study data. The interaction study data confirmed the inert nature of the carrier. PGS was found to be a better carrier than SSG, in enhancing the poor aqueous solubility of the model drug. Therefore, it can be concluded that the aqueous solubility of poorly soluble drugs can be significantly improved by utilizing the solid dispersion technique, relatively easily.
